# MUC1/CA15-3 identifies a clear cell renal carcinoma characterized by Sunitinib response with a specific metabolic signature

**DOI:** 10.1007/s10238-026-02042-5

**Published:** 2026-01-14

**Authors:** Giuseppe Lucarelli, Francesco Lasorsa, Martina Milella, Antonio d’Amati, Giuseppe Ingravallo, Antonio Di Bari, Savio Domenico Pandolfo, Roberto Tamma, Michela De Giorgis, Domenico Ribatti, Annalisa Schirinzi, Francesca di Serio, Alessandro Caniglia, Francesco Alfredo Zito, Emanuele Naglieri, Michele Battaglia, Pasquale Ditonno, Monica Rutigliano

**Affiliations:** 1 Urology and Kidney Transplantation Unit, Department of Precision and Regenerative Medicine and Ionian Area, University of Bari “Aldo Moro”, Bari, 70124 Italy; 2 Urology Unit, IRCCS Istituto Tumori “Giovanni Paolo II”, Bari, 70124 Italy; 3 Pathology Unit, Department of Precision and Regenerative Medicine and Ionian Area, University of Bari “Aldo Moro”, Bari, 70124 Italy; 4 Department of Neurosciences, Science of Reproduction and Odontostomatology, Federico II University, Naples, 80138 Italy; 5 Department of Translational Biomedicine and Neuroscience, University of Bari “Aldo Moro”, Bari, 70124 Italy; 6 Clinical Pathology Unit, Polyclinic University Hospital, Bari, 70124 Italy; 7 Pathology Unit, IRCCS Istituto Tumori “Giovanni Paolo II”, Bari, 70124 Italy; 8 Oncology Unit, IRCCS Istituto Tumori “Giovanni Paolo II”, Bari, 70124 Italy; 9 Department of Precision and Regenerative Medicine and Ionian Area - Urology, Andrology and Kidney Transplantation Unit, University of Bari “Aldo Moro”, Bari, Italy

**Keywords:** MUC1, CA15-3, Renal cell carcinoma, Sunitinib, Angiogenesis

## Abstract

Clear cell renal carcinoma (ccRCC) is a prevalent kidney cancer with limited effective biomarkers for prognosis and treatment guidance. Despite advancements, a significant portion of ccRCC cases progress to advanced stages, necessitating novel diagnostic tools. Here, we investigated the expression of MUC1 and its soluble form, CA15-3, in ccRCC, evaluating their potential as biomarkers for angiogenesis and response to sunitinib therapy. Molecular analyses showed that MUC1 expression was associated with angiogenesis, epithelial–mesenchymal transition, hypoxia/metabolism regulation, and complement system activation. In particular MUC1 overexpression correlated with increased microvascular density in vitro and in vivo models. Elevated CA15-3 levels were associated with tumor burden and predict clinical response to sunitinib in metastatic ccRCC. Metabolomic analysis showed that sunitinib-responding tumors were characterized by specific metabolic changes involving glucose and lipid metabolism, in association with impaired oxidative phosphorylation. MUC1 expressing ccRCC is a high angiogenic tumor that presents characteristics of increased aggressiveness, and a specific metabolic profile. Serum CA15-3 is a marker of poor survival and predicts response of sunitinib in patients with metastatic disease.

**Supplementary Information:**

The online version contains supplementary material available at 10.1007/s10238-026-02042-5.

## Introduction

 Clear cell renal cell carcinoma (ccRCC) is the most common malignant tumor of kidney, and one of the ten most frequent cancers in adult population. The American Cancer Society estimates that in 2025 in the United States, 80,980 new cases will be diagnosed and 14,510 patients will die from kidney cancer [1]. Although RCC is diagnosed early today, up to 30% of cases are in a locally advanced or metastatic stage, and about one third of patients undergoing surgery with a curative intent, will experience recurrence or distant metastases [2]. Therefore, the identification of diagnostic and prognostic factors has a fundamental role in this disease, considering that to date we have no effective molecular marker that may help to guide treatment.

A prognostic role has been proposed for several molecules associated with different features of RCC biology, including serum and histological markers [3, 4].

Recent evidence demonstrates that RCC cells undergo extensive metabolic reprogramming, including glycolysis, lipid biosynthesis and catabolism, and amino acid metabolism [5–7]. Such metabolic rearrangements are largely driven by the dysregulated expression and activity of key metabolic enzymes and regulatory factors, which are tightly associated with tumor aggressiveness, therapeutic resistance, and adverse clinical outcomes. For example, activation of the kynurenine pathway (a metabolite derived from tryptophan) promotes the survival, migration, and chemoresistance of renal cancer cells, and the kynurenine/tryptophan ratio serves as a prognostic marker for cancer-specific survival and progression-free survival [8].

Mucins are extensively glycosylated proteins that are typically expressed on the apical surface of epithelial cells. They are classified into two main categories, secreted and transmembrane mucins, and their aberrant overexpression contributes to oncogenesis by promoting receptor tyrosine kinase signalling, loss of epithelial cell polarity, constitutive activation of growth and survival pathways [9].

In a recent study we showed that mucin 1 (MUC1), a transmembrane O-glycosylated protein, is overexpressed in a subset of ccRCC characterized by poor prognosis [5]. In cancer cells, MUC1 induces metabolic reprogramming, modulates the immunoflogosis, and promotes metastatic progression [10–12].

CA15-3 (Cancer Antigen 15 − 3) is the soluble form of MUC1, it is detectable in serum and is the most widely used tumor marker for breast cancer [13]. Elevated values of this biomarker can be associated with tumors other than breast cancer (including pancreas, lung and ovarian cancer) as well as with non-neoplastic conditions such as endometriosis, tuberculosis, liver diseases and other inflammatory disorders. The diagnostic and prognostic role of this biomarker in ccRCC is poorly understood.

Metastatic RCC is one of the most chemotherapy resistant malignancies, and is associated with a poor clinical outcome (especially for grade 4 tumors with sarcomatoid component) [14–16]. The introduction into clinical practice of drugs such as tyrosine kinase inhibitors (TKIs) and, more recently, immune checkpoint inhibitors (ICIs), has radically changed the prognosis of these patients [17]. In this scenario, the identification of predictive biomarkers for treatment response is of utmost importance.

In this study, we evaluated the MUC1 gene expression using the microarray datasets deposited in the public functional genomics data repository Gene Expression Omnibus (GEO) and investigated the biological processes associated with MUC1 expression in ccRCC.

In addition, we studied the tissue expression of MUC1 in ccRCC samples, evaluated the association between serum CA15-3 and clinicopathological characteristics and investigated its prognostic role of in a large cohort of patients with localized or locally advanced ccRCC. Finally, we analyzed the role of this biomarker as predictor of response in a group of patients with metastatic ccRCC, undergoing treatment with sunitinib, and identified the metabolic characteristics associated to therapeutic response.

## Methods

### Single-Cell RNA-Sequencing analysis (scRNA-seq)

The analysis of scRNA-seq data was performed using Tumor Immune Single-cell Hub 2 (TISCH2) online tool, which provides a detailed cell-type annotation at the single-cell level [18]. MUC1 expression was evaluated in two ccRCC datasets (GSE159115 and GSE171306) [19, 20].

In addition, we used data from the Cancer Single-cell State Atlas (CancerSEA) database (http://biocc.hrbmu.edu.cn/CancerSEA/) to explore how MUC1 was associated to distinct functional states of renal cancer cells at the single-cell level [21].

## Gene set enrichment analysis (GSEA)

Gene expression data from the GSE15641 and KIRC-TCGA databases were downloaded [22, 23]. For each cohort, we stratified the patients by MUC1, into the top 50% and bottom 50%. Next, we generated a rank file for each expressed gene by the log2 fold change of high MUC1 (MUC1^H^) samples over low MUC1 (MUC1^L^) samples. GSEA was run against the HALLMARK pathway collection, as compiled by Molecular Signature Database (MSigDB) [24]. The normalized enrichment score (NES) was used to evaluate the extent and direction of enrichment of each pathway. A P-value of < 0.05 was considered statistically significant. Moreover, to define the ccRCC hallmark fingerprint related to MUC1 expression according to Hanahan and Weinberg conceptual framework [25], we used a platform accessible at www.cancerhallmarks.com [26].

## Evaluation of MUC1 prognostic role in KIRC-TCGA database

MUC1 expression data and gene copy number alterations (CNAs) for KIRC-TCGA database were downloaded from UCSC Xena (https://xena.ucsc.edu/) [27]. The prognostic role of MUC1 alterations in terms of overall survival (OS), cancer specific survival (CSS), and progression-free survival (PFS) were estimated using the Kaplan-Meier method.

## Primary cell cultures from tumor renal tissues

Renal cancer tissue specimens were immediately placed in a Petri dish with phosphate-buffered saline (PBS) 1x and cut into small pieces of about 1 mm^3^. Each small piece of tissue was placed on the surface of the Petri dish with Dulbecco’s Modified Eagle Medium (DMEM, Invitrogen, Life Technologies, Monza, Italy) supplemented with 10% fetal bovine serum (FBS, Sigma-Aldrich, Milan, Italy) and 1% penicillin-streptomycin-L-glutamine (Sigma-Aldrich, Milan, Italy). Cell proliferation was obtained around the kidney specimens as previously described [28]. Kidney epithelial neoplastic cells were isolated with EpCAM (CD326) Ab-conjugated magnetic microbeads (Miltenyi Biotec, BergischGladbach, Germany) under the effect of a magnetic field generated by the Mini MACS Separation Unit (MiltenyiBiotec). These cells were then characterized for EpCAM, CA IX, and MUC1 by immunocytochemistry (Supplementary Fig. 1). Primary ccRCC cells were used for proliferation studies at the second passage.

## Immunohistochemistry and tissue microarray construction

High-density tissue microarrays were used for MUC1 and CD31 immunostaining. Archived formalin-fixed paraffin-embedded nephrectomy tissue samples for 100 primary ccRCC were obtained.

All tumor cores were identified by two uro-pathologists. These were selected by identifying representative tumor-containing slides and were used to assign the original tumor grade in each case. Three-mm cores were removed from the selected area (region of interest) using a needle punch. These 3-mm donor cores were subsequently embedded in previously arranged recipient paraffin blocks through a precisely spaced 15-hole array pattern. Core positions in the recipient paraffin block were noted on a TMA map. After paraffin cooling, the recipient blocks were cut in the microtome and used for immunohistochemistry. TMA were deparaffinized and rehydrated with xylene and graded alcohol series. Slides were subjected to specific epitope unmasking by microwave treatment (700 W) in citrate buffer (0.01 M pH = 6.0). After antigen retrieval, TMAs were incubated for 10 min with 3% H_2_O_2_ to block endogenous peroxidase activity. Sections were treated with serum-free protein block (Dako, Agilent, Glostrup, Denmark) at room temperature (RT) for 10 min and then incubated with anti-MUC1 antibody (1:200, NCL-L-EMA, Novocastra Laboratories Ltd, Newcastle, UK) and anti-CD31 (PECAM-1) antibody (1:100, NCL-CD31-1A10, Novocastra Laboratories Ltd, Newcastle, UK) at 4 °C overnight. Binding of the secondary biotinylated antibody was detected using the Dako Real EnVision Detection System, Peroxidase/DAB kit (Dako, Agilent, Glostrup, Denmark), according to the manufacturer’s instructions. Sections were counterstained with Mayer’s hematoxylin (blue) and mounted with glycerol (Dako Cytomation). Negative controls were obtained by incubating serial sections with the blocking solution and then omitting the primary antibodies.

Sections from both experimental groups (MUC1^H^ and MUC1^L^), were scanned using the whole-slide morphometric analysis scanning platform Nano Zoomer S60v2MD (Hamamatsu Photonics K.K., EU). Digital slides were inspected with Aperio ImageScope v.11 software (Leica Biosystems, Nussloch, Germany) at 20X magnification and 10 fields with an equal area were selected for the analysis. Specific staining was quantified using the Nikon NIS-Elements Advanced Research version 6.01 software (Nikon Instruments, Tokyo, Japan) and expressed as percentage of positive pixels in the analyzed area.

### Immunofluorescence microscopy

A double-label immunofluorescence was performed to evaluate the expression of MUC1, CD31, PTX-3, C3, Vimentin and Snail + Slung. The following primary antibodies were used: anti-MUC1 antibody (mouse, 1:200, NCL-L-EMA, Novocastra Laboratories Ltd, Newcastle, UK), anti-CD31 (rabbit, 1:50, NBP1-71663, Novus Biologicals, Bio-Techne Ltd., Abingdon, U.K.), anti-PTX-3 (mouse, 1:100, sc-373951, Santa Cruz Biotechnology, Santa Cruz, CA), anti-C3 (rabbit, 1:100, ab97462, Abcam, Cambridge, U.K.), anti-Vimentin (mouse, ready to use, GA63061-2, Dako, Agilent, Glostrup, Denmark) and anti-Snail + Slung (rabbit, 1:100, ab180714, Abcam, Cambridge, U.K.).

Initially, MUC1 and CD31 protein expression and localization were assessed on paraffin-embedded tissue sections of kidney specimens.

After antigen unmasking, the tissue sections were blocked with 2% BSA in PBS for 1 h at room temperature. Sections were incubated at 4 °C overnight with a primary antibody against MUC1 in blocking, followed by incubation for 1 h at room temperature with the secondary antibody Alexa Fluor 555 goat anti-mouse IgG (1:200; ab150114, Abcam, Cambridge, U.K.). Sections were washed in PBS and blocked with 2% BSA for 1 h at room temperature, then incubated overnight at 4 °C with primary antibodies against CD31 in blocking followed by incubation for 1 h with the secondary antibody Alexa Fluor 488 goat anti-rabbit IgG (1:500; ab150077, Abcam, Cambridge, U.K.).

PTX3 and C3 protein expression was evaluated on the same paraffin-embedded kidney tissue sections from both MUC1^H^ and MUC1^L^ groups.

After antigen unmasking, the tissue sections were blocked with 5% Goat Serum in PBS for 1 h at room temperature. Sections were incubated at 4 °C overnight with a primary antibody against PTX-3 in blocking, followed by incubation for 1 h at room temperature with the secondary antibody Alexa Fluor 555 goat anti-mouse IgG (1:200; ab150114, Abcam, Cambridge, U.K.). Sections were washed in PBS and blocked with 1% BSA for 1 h at room temperature, then incubated overnight at 4 °C with primary antibodies against C3 in blocking followed by incubation for 1 h with the secondary antibody Alexa Fluor 488 goat anti-rabbit IgG (1:500; ab150077, Abcam, Cambridge, U.K.).

The evaluation of Vimentin and Snail + Slung protein expression was carried out on same paraffin-embedded tissue sections.

After antigen unmasking, the tissue sections were blocked with 1% BSA in PBS for 1 h at room temperature. Sections were incubated at 4 °C overnight with a primary antibody against Vimentin, followed by incubation for 1 h at room temperature with the secondary antibody Alexa Fluor 555 goat anti-mouse IgG (1:200; ab150114, Abcam, Cambridge, U.K.). Sections were washed in PBS and blocked with 2% BSA for 1 h at room temperature, then incubated overnight at 4 °C with primary antibodies against Snail + Slung in blocking followed by incubation for 1 h with the secondary antibody Alexa Fluor 488 goat anti-rabbit IgG (1:500; ab150077, Abcam, Cambridge, U.K.).

To stain the nuclei, after washing in PBS, the sections were incubated with 4′,6-diamidino-2-phenylindole (DAPI) diluted 1:6000 in PBS (Sigma‒Aldrich, St. Louis, USA). The slides were mounted in Fluoromount (Dako Cytomation) and sealed.

Tumor renal cells (ccRCC cells) of MUC1^H^ and MUC1^L^ groups were seeded at a density of 2 × 10^5^ on glass coverslips and left to adhere overnight at 37 °C in 5% CO_2_. These preparations were double-stained for NDUFA4L2 (Proteintech, Chicago, IL, USA) and MitoTracker™ Red CMXRos (Molecular Probes, Life Technologies, Monza, Italy). The expression and localization of proteins was evaluated by immunofuorescence microscopy analysis. Mitocondrial mass was determined by incubating the live cells with 500 nM MitoTracker™ Red CMXRos in growth medium, for 20 min at 37 °C. The cells were fixed using ice-cold 4% paraformaldehyde for 10 min at room temperature. Then the preparations were blocked with 1% BSA in PBS for 1 h at room temperature and incubated overnight at 4 °C with a primary antibody against NDUFA4L2 (1:25 in blocking) followed by incubation for 1 h at 37 °C with the secondary antibody goat anti-rabbit IgG FITC (1:200; Novus Biologicals). All preparations were counterstained with DAPI (Sigma‒Aldrich, St. Louis, USA). Negative controls were performed by omitting the primary antibodies. Specific fluorescence was acquired by Nikon Eclipse Ci-L Plus microscope (Nikon Instruments, Tokyo, Japan) using an ×20 objective lens. Specific staining was quantified using the Nikon NIS-Elements Advanced Research version 6.01 software (Nikon Instruments, Tokyo, Japan) according to the manufacturer’s guidelines and expressed as percentage of positive pixels in the analyzed area.

## Drug sensitivity analysis

Sunitinib sensitivity analysis was performed using UCSCXenaShiny tool (https://github.com/openbiox/UCSCXenaShiny) [29]. We used the pharmacogenomics modules based on six publicly accessible pharmacogenomics studies (GDSC1 and GDSC2, CTRP1 and CTRP2, PRISM, gCSI), to explore the sensitivity of cancer cell lines to sunitinib on the basis of MUC1 expression.

## Cell viability assay

Cell viability after exposure to 5µM of sunitinib (HY-10255 A; MedChemExpress) was assayed using the trypan blue dye exclusion and 3-(4,5-dimethylthiazol-2-yl)−2,5-diphenyltetrazolium bromide (MTT) assay. For the dye exclusion test and MTT assay, tumor cells of MUC1^H^ and MUC1^L^ groups were seeded at a density of 1.5 × 10^5^ and 1.5 × 10^4^ cells in six-well and 96-well plates, respectively (Sigma Aldrich, Milan, Italy) and incubated overnight at 37 °C, 5% CO_2_. In the first part of the experiment, the cells were exposed to 5µM of sunitinib for 24 h or incubated in medium alone. After several washes to remove sunitinib the cells were again incubated in medium alone for 24 h and 48 h. Each experimental condition was performed in triplicate.

### Real-Time PCR

Total RNA of normal and tumor tissues was reverse transcribed with the High-Capacity cDNA Reverse Transcription Kit (Applied Biosystems Foster City, CA, USA), following the manufacturer’s instructions. Quantitative real-time polymerase chain reactions (PCR) were performed using the iQTM SYBR Green Supermix buffer (6mMMgCl2, dNTPs, iTaq DNA polymerase, SYBR Green I, fluorescein and stabilizers) (BIO-RAD Laboratories, Hercules, CA, USA). The primers used for rea-time PCR are listed in Supplementary Table 1.

Quantification of the mRNA levels was performed on a MiniOpticon Real-Time PCR detection system (BIO-RAD Laboratories). In the PCR reactions, the following protocol was used: polymerase activation at 95 °C for 3 min, followed by 45 cycles at 95 °C for 10 s, 60 °C for 30 s. Melting curves were generated through 60 additional cycles (65 °C for 5 s with an increment of 0.5 °C/cycle). Gene expression results were obtained as mean Ct (threshold cycle) values of triplicate samples. Expression was determined using the 2-ΔΔCt method. Expression values were normalized to β-Actin.

### Small interfering RNA (siRNA) transfection

Isolated MUC1^H^ tumor renal cells were cultured at 2 × 10^5^ cells per well in a 12-well plate with Keratinocyte Serum-Free Medium (KSFM), supplemented with 5 ng/ml recombinant epidermal growth factor (rEGF), 50 µg/ml bovine pituitary extract (BPE) (Gibco, Life Technologies, Monza, Italy) and 30 ng/ml cholera toxin (Sigma-Aldrich, Milan, Italy). The transfection of siRNA was carried out using Lipofectamine 3000 (Life Technologies, Monza, Italy) in accordance with the manufacturer’s procedure. For each transfection, 50 nM of small interfering RNA targeting MUC1 (siMUC1) (Qiagen, Hilden, Germania) were used. In transfection experiments, a mock-transfection control was performed by putting cells through the transfection procedure without adding siRNA. The validated non-silencing siRNA sequence AllStars Negative Control siRNA (50 nM, Qiagen Hilden, Germania) was used as negative control (Supplementary Fig. 2). Each transfection experiment was performed in triplicate. After transfection, normal and tumor renal cells were incubated for 12 h and 24 h at 37 °C in 5% CO2 and used for total RNA extraction, measurement of glucose consumption and lactate production and cell viability assays.

### Measurement of glucose consumption and lactate production

5 × 10⁴ cells were seeded in culture dishes. Following a 6-hour incubation, the medium was replaced with fresh serum-free DMEM. Cells were then incubated for 12–16 h, after which the medium was collected to measure glucose and lactate concentrations. Glucose levels were determined using the Glucose (GO) Assay Kit (Sigma‒Aldrich, St. Louis, USA), and lactate levels were measured using the Lactate Assay Kit (Sigma‒Aldrich, St. Louis, USA).

### In vivo Chorioallantoic membrane (CAM) angiogenic assay

Fertilized White Leghorn/Isa Brown chicken eggs obtained from a commercial supplier staged according to Hamburger and Hamilton (HH) will be placed, at the onset, into an incubator and kept under constant humidity at 37 °C (day (D) 0). At stage HH3 (D3), a square window was opened into the eggshell after the removal of 2–3 ml of albumen so that the developing chorioallantoic membrane was detached from the shell itself and the underlying CAM vessels were observed. The window was sealed with a glass coverslip and the eggs returned to the incubator until the day of experiment. At D10 of CAM development the top of the growing CAMs will be visible, and its endothelium exhibits an intrinsically high mitotic rate. The coverslips were removed, and a 1mm^3^sterilized gelatin sponge was implanted onto the CAM [30]. Three replicates of 1.5 × 10^6^ of each cell population (MUC1^H^, MUC1^L^, MUC1^H^ + sunitinib, and MUC1^L^ + sunitinib) were re-suspended in 2 µl PBS and then released onto the gelatin sponge. The same volume of PBS as well as the same volume of sunitinib at 5 µM were used as negative controls. The transplanted eggs returned to the incubator for the next 4 days. At D14 the eggs were used for experimental determinations. Gelatin sponge with surrounding CAM were fixed with 4% paraformaldehyde for 24 h. CAMs were examined at D14 and photographed with a stereo microscope equipped with a digital camera. The angiogenic response was evaluated by the IKOSA CAM Assay Application image analyzer system as total vascular area and number of vessels branching points.

### Metabolomics

Primary renal tumors were collected from patients who underwent radical or partial nephrectomy for metastastic ccRCC. MUC1 expression was evaluated by immunohistochemistry stratifying samples according to staining intensity as tumor with high MUC1 expression (MUC1^H^; ≤ 50% positive tumor cells), tumor with low MUC1 expression (MUC1^L^; 50 − 20% positive tumor cells), and tumor that do not express MUC1 (MUC1^neg^). In addition, samples were further stratified based on patient response to sunitinib [complete response (CR) vs. progressive disease (PD)], and the following groups were analyzed: MUC1^H^-CR (*n* = 10), MUC1^L^-PD (*n* = 10), and MUC1^neg^-PD.

All tissue samples were maintained at − 80 °C until processed and prepared for the appropriate investigation, liquid chromatography/mass spectrometry or gas chromatography/mass spectrometry, as previously described [10, 15].

Global biochemical profiles were determined in tumor samples and compared across groups. Comparisons of metabolites median values between different groups were evaluated by Mann–Whitney U test. MetaboAnalyst 6.0 (https://www.metaboanalyst.ca) was used for clustering analysis, heatmap generation and principal component analysis (PCA).

Additional details regarding the experimental procedures are provided in the Supplementary Materials and Methods section.

### Study population

#### Non-metastatic patients

Serum CA 15 − 3 (normal range: 0–25 U/mL), was prospectively measured in a cohort of 914 consecutive patients who underwent radical or partial nephrectomy for localized or locally advanced clear cell RCC (ccRCC) at our institution from January 2016 to December 2023 and in 300 healthy adult volunteers with no evidence of malignancy. Patients with visceral metastases at diagnosis were excluded. Patients with an estimated glomerular filtration rate (eGFR calculated using MDRD equation) < 60 mL/min/1.73 m^2^ and with other known causes of elevated levels of this biomarker were excluded from the study (namely, patients with other malignant tumors, breast and liver diseases, endometriosis, sarcoidosis, tuberculosis, and systemic lupus erythematosus). All patients were preoperatively staged by thoraco-abdominal Computed Tomography (CT) or Magnetic Resonance Imaging (MRI). Tumor staging was reassigned according to the seventh edition of the AJCC-UICC TNM classification. Tumor grade on pathological tissues was attributed with hematoxylin–eosin (HE) staining in according to 2022 WHO/ISUP grading system.

Measurement of serum CA15-3 was performed with electrochemiluminescence immunoassay (ECLIA) on a fully automated Roche Cobas 8000 analyzer (Roche Diagnostics GmbH, Mannheim, Germany).

### Metastatic patients

To study the role of CA15-3 as biomarker of response to antiangiogenic therapy, a total of 48 patients with metastatic ccRCC, who received sunitinib were retrospectively enrolled.

Serum CA 15 − 3 was measured before therapy administration and re-evaluated after 24 weeks of treatment. Patients included in the analysis had visceral metastases with measurable lesions (i.e., a maximum transverse diameter ≥ 1 cm on CT or MRI). In addition, they must have undergone a clinical efficacy evaluation using the Response Evaluation Criteria in Solid Tumors version 1.1 (RECIST1.1) [31]; and have a performance status (PS) score ≤ 2. Patients who discontinued treatment due to grade 3–4 immune-related adverse reactions, or with additional primary tumors other than ccRCC were excluded.

Complete response (CR) was defined as the disappearance of all target lesions with a short-axis lymph node diameter < 10 mm. Partial response (PR) was defined as a reduction of ≥ 30% in the sum of the longest diameters of all target lesions. Progressive disease (PD) was defined as an increase of at least 20% in the sum of the longest diameters of target lesions with an absolute increase of at least 5 mm, and the appearance of one or more new lesions. Stable disease (SD) was defined as tumor shrinkage of less than 30% and an increase of less than 20% between the PR and PD. ΔCA 15 − 3 was defined as the ratio of pre-treatment to post-treatment CA15-3 values.

### Statistical analysis

Comparisons of median values between different groups were evaluated by Mann–Whitney U test. Continuous variables were summarized with median and 95% confidence interval for median (95% CI). Categorical variables were summarized with frequency counts and percentages.

Spearman test was applied to evaluate the correlations between the CA15-3 serum values and tumor stage/diameter.

CA 15 − 3 cut-off values were determined using Receiver Operating Characteristic (ROC) analysis and quantified in terms of Area under the Curve (AUC) and corresponding 95% confidence interval (95% CI) (Supplementary Fig. 3).

In the CSS analysis, patients who died of RCC-unrelated causes or were lost to follow-up were censored. Recurrence-free survival (RFS) was calculated from the date of surgery to the date of disease recurrence. Disease recurrence/progression was assessed radiographically (using CT scan or MRI) with a surveillance schedule based on EAU guidelines.

In metastatic population, time to second progression (PFS2) was calculated. PFS2 was defined as the time from the start of first-line therapy to objective radiological tumor progression on next-line treatment. Estimates of CSS, RFS, and PFS2 were calculated according to the Kaplan–Meier method and compared with the log-rank test.

Patients without a tumor progression to following line of treatment or death or lost at follow-up at the time of analysis were censored at their last follow-up date.

Multivariable analysis was performed using the Cox proportional hazards regression models to identify the most significant variables for predicting CSS, RFS, and PFS2. A backward selection procedure was performed with removal criterion *P* > 0.10 based on likelihood ratio tests. A P-value of < 0.05 was considered statistically significant.

Statistical tests were performed using MedCalc 19.6.3 (MedCalc software, Mariakerke, Belgium) and PASW 18 software (PASW 18, SPSS, Chicago, IL, USA).

## Results

### Expression of MUC1 at the single-cell level

**Fig. 1:**
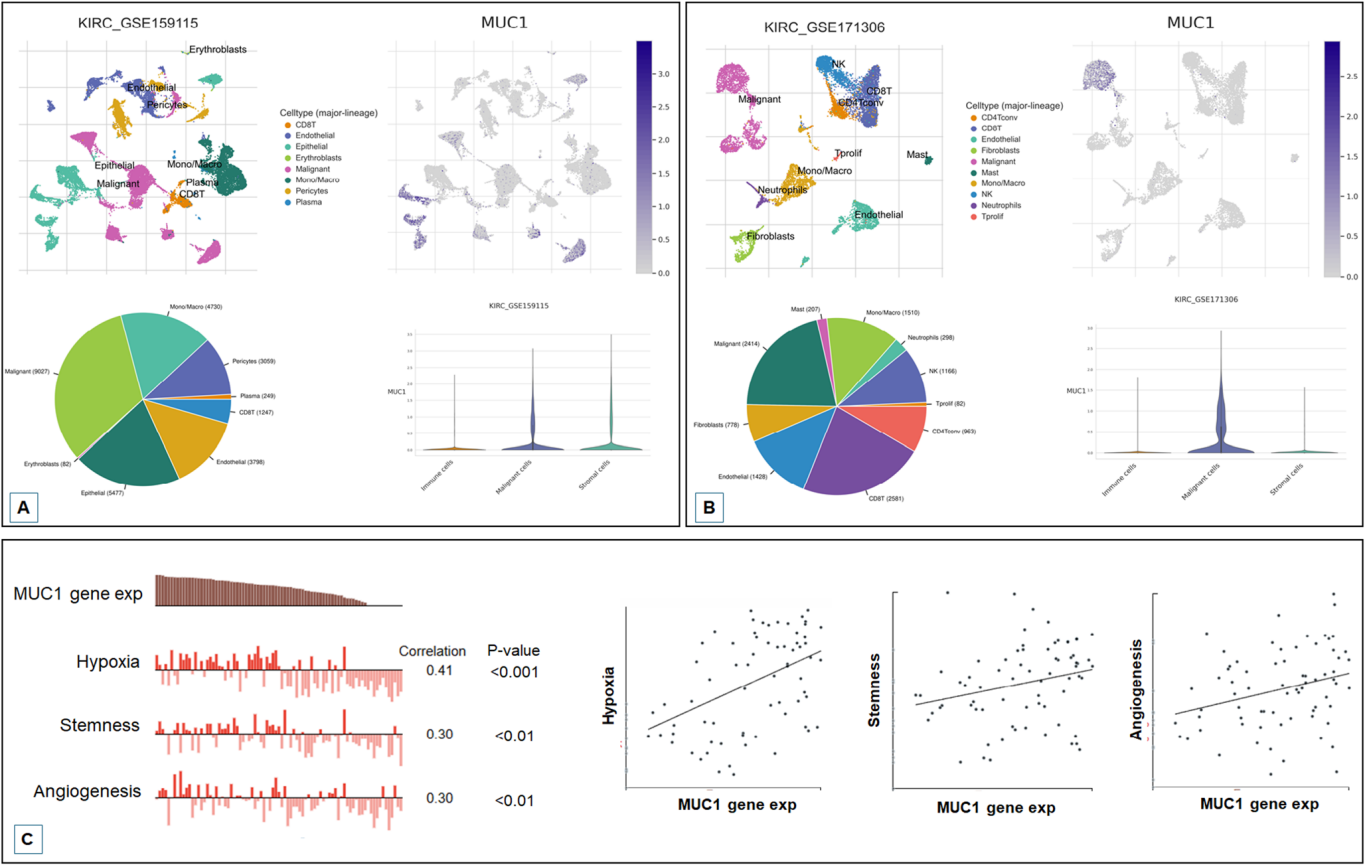
Expression of MUC1 in ccRCC cells was evaluated by TISCH2 in GSE159115 (**A**) and GSE171307 (**B**) datasets. CancerSEA database was used to explore how MUC1 was associated to distinct functional states of renal cancer cells at the single-cell level (**C**)

The evaluation of MUC1 expression in ccRCC across different cell subpopulations was performed using the TISCH2 single-cell database. The analysis revealed that MUC1 was primarily expressed in malignant cells (Fig. 1A and B). Moreover, the functional states correlation analysis using cancerSEA, showed that MUC1 exhibited a positive correlation with hypoxia, stemness, angiogenesis and proliferation (Fig. 1C).

### Hallmark cancer pathways are associated with high MUC1 expression

**Fig. 2:**
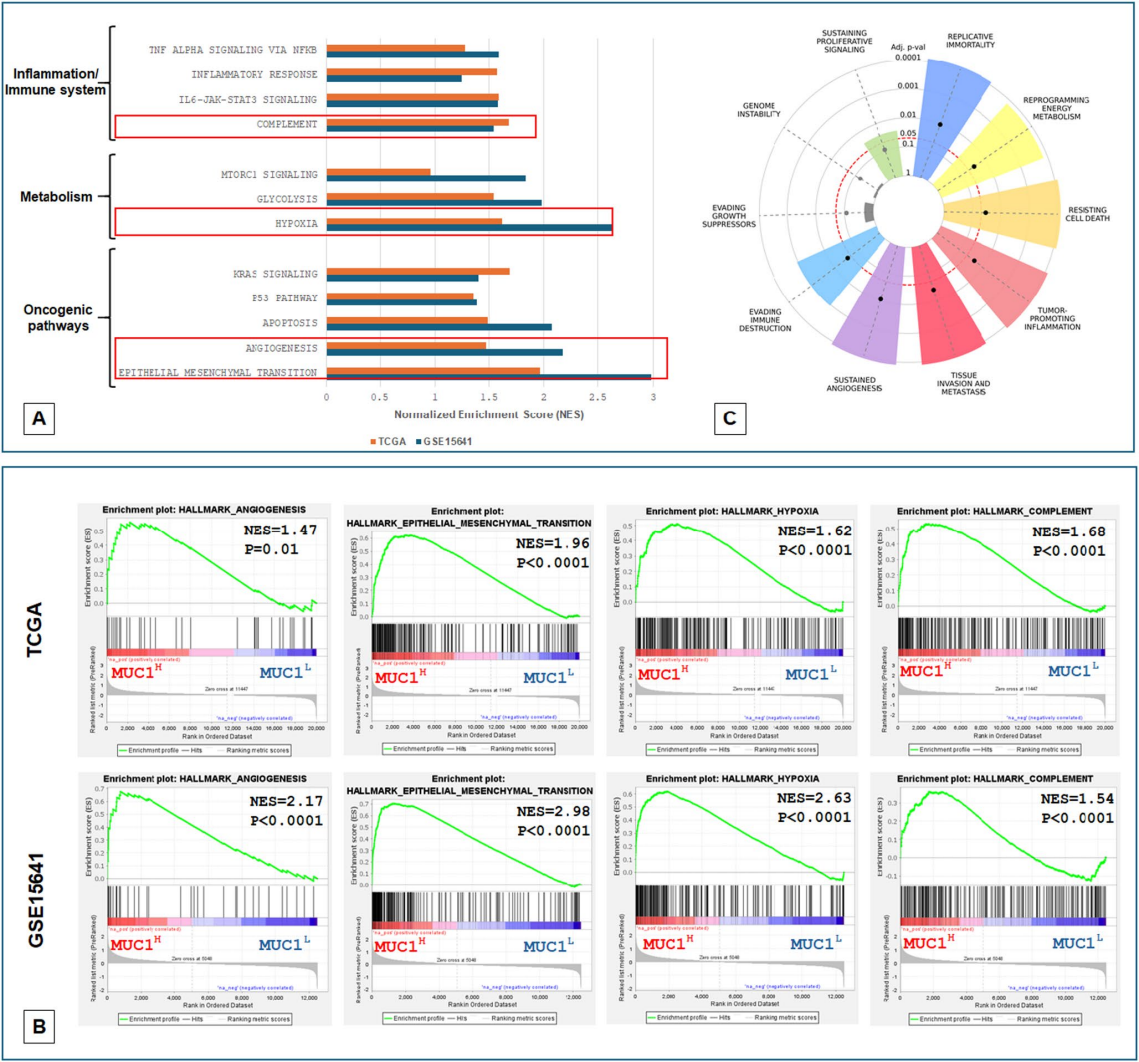
GSEA showed that genes associated with MUC1 upregulation were significantly enriched in metabolic, oncogenic, and inflammation pathways in TCGA and GSE15641 datasets (**A**-**B**). Hallmark enrichment plot for MUC1^H^ tumors (**C**)

GSEA revealed that genes associated with MUC1 upregulation were significantly enriched in metabolic, oncogenic, and inflammation pathways. In particular MUC1^H^ ccRCC featured multiple enriched gene sets depicting angiogenesis, epithelial–mesenchymal transition (EMT), hypoxia/metabolism, and complement system activation (Fig. 2A and B). The hallmark enrichment plot (Fig. 2C) summarizes the results of GSEA for the cancer hallmarks in MUC1^H^ tumors.

#### MUC1^H^ CcRCC shows modulation of different cancer pathways

To validate the CancerSEA and GSEA results, we studied the tissue expression of the proteins involved in the over-represented pathways.

**Fig. 3:**
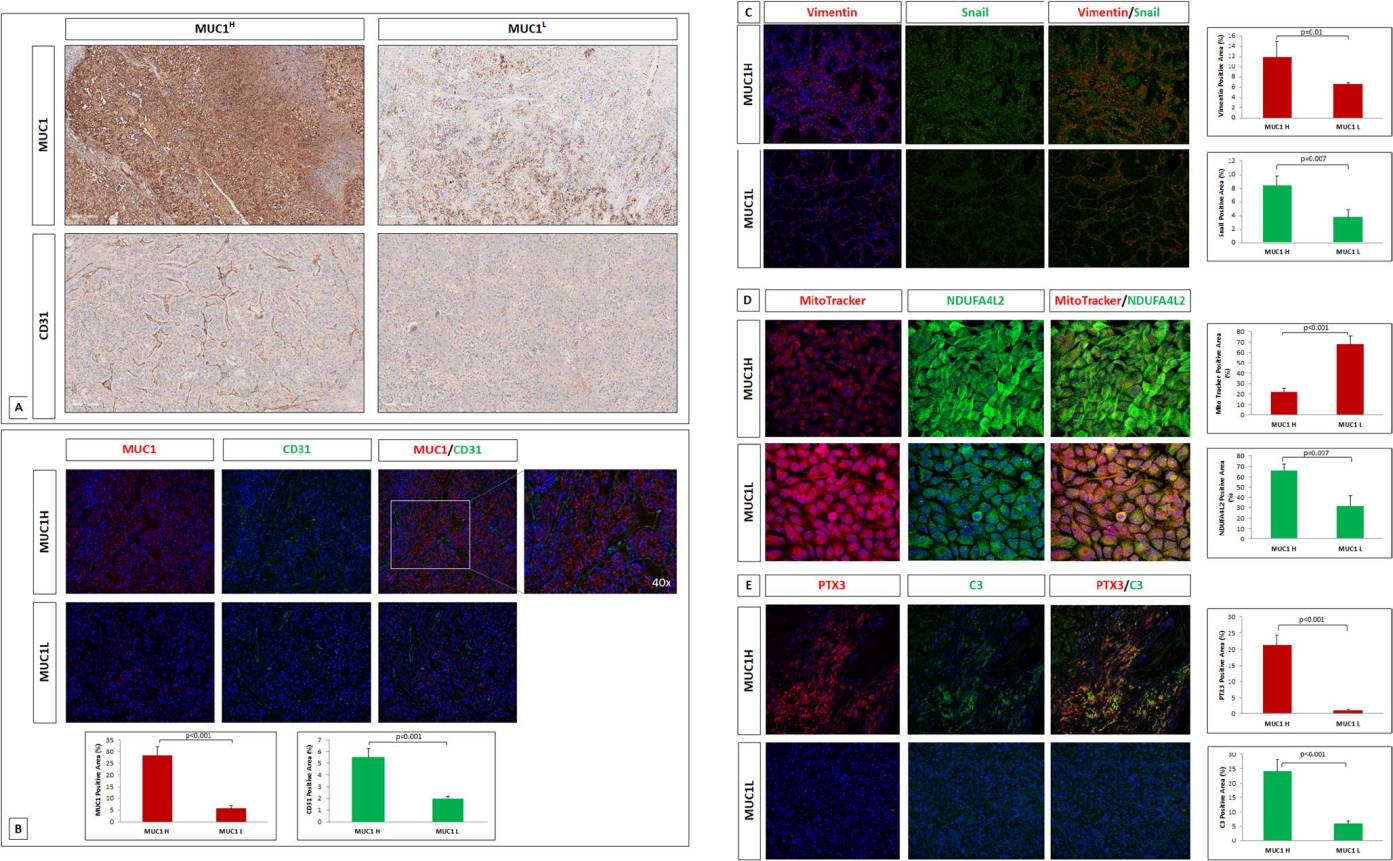
Association between MUC1 and CD31 expressions assessed by immunohistochemistry (**A**) and immunofluorescence (**B**). Representative images and quantification of immunofluorescence for vimentin/Snail (**C**), MItoTracker/NDUFA4L2 (**D**), and PTX3/C3 (**E**) according to MUC1 expression

To investigate tumor angiogenesis, we evaluated the microvessel density (MVD) by expression of CD31 in MUC1^H^ versus MUC1^L^ human ccRCC specimens, using immunohistochemistry and immunofluorescence. MUC1 was highly expressed in 38% of cases with a prevalently cytoplasmic/membranous pattern. In addition, we observed an increased expression of CD31 in MUC1^H^ samples (Fig. 3A). These findings were confirmed by immunofluorescence that showed co-expression of MUC1 and CD31 (Fig. 3B).

Next, we investigated the activation of additional oncogenic programs in MUC1^H^ tumors, based on the results of the GSEA.

In particular, MUC1^H^ renal tumors displayed the activation of EMT program as shown by increased expression of vimentin and Snail transcription factor in tissue samples (Fig. 3C). We then explored the metabolic reprogramming/hypoxia hallmark, evaluating the expression of NDUFA4L2 and the mitochondrial mass in primary cancer cell lines. MUC1 expressing cells exhibited increased levels of NDUFA4L2 and a reduced number of mitochondria (Fig. 3D), in accordance with our previous results [10]. Finally, to study the activation of complement system in association with MUC1 expression, we evaluated the deposition of PTX3 and C3 in cancer tissue. Interestingly, C3 expression was markedly increased in MUC1^H^ ccRCC tissue samples and colocalized with PTX3 (Fig. 3E).

### MUC1 expression in renal cancer primary cells is associated with aggressive features but increased sensitivity to sunitinib

In silico analysis suggested that MUC1 expression was associated with increased sensitivity of cancer cells to sunitinib (Supplementary Fig. 4).

**Fig. 4:**
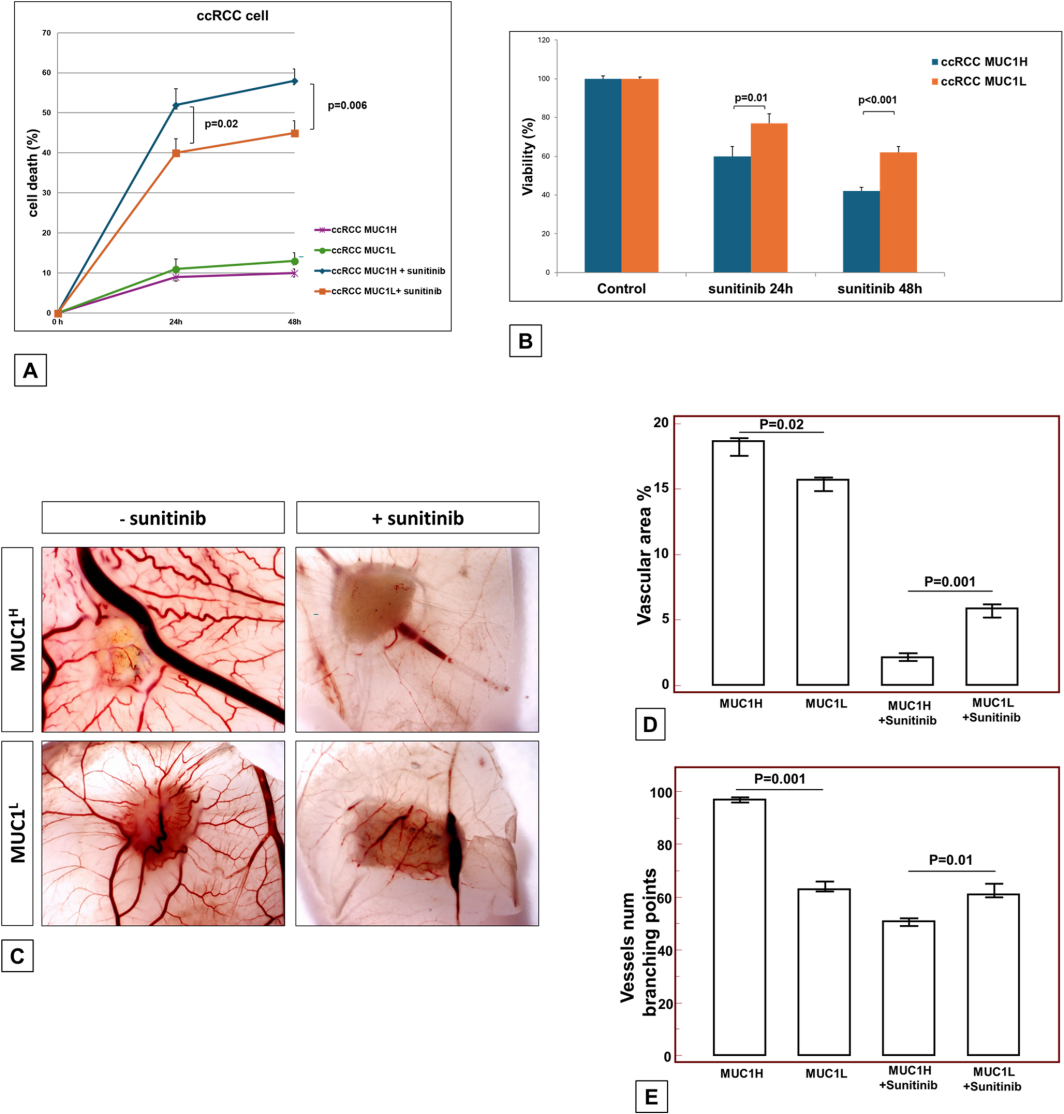
Death rate (**A**) and MTT assay (**B**) of MUC1^H^ and MUC1^L^ tumor cells pre-treated with sunitinib. Chorioallantoic membrane angiogenic assay for MUC1H and MUC1L tumor cells pre-treated or not with sunitinib (**C**). The angiogenic response was evaluated as total vascular area (**D**) and number of vessels branching points (**E**)

To gain further insight into the role of MUC1 in sunitinib sensitivity, we evaluated the death rate of primary human cccRCC cells treated with this drug. The death rate of treated MUC1^H^ tumor cells was significantly higher than that of treated MUC1^L^ cells (*P* = 0.006; Fig. 4A). The MTT assay confirmed these results, demonstrating significant differences in cell viability when MUC1^H^ and MUC1^L^ tumor cells were pre-treated with sunitinib (*P* < 0.001; Fig. 4B).

**Fig. 5:**
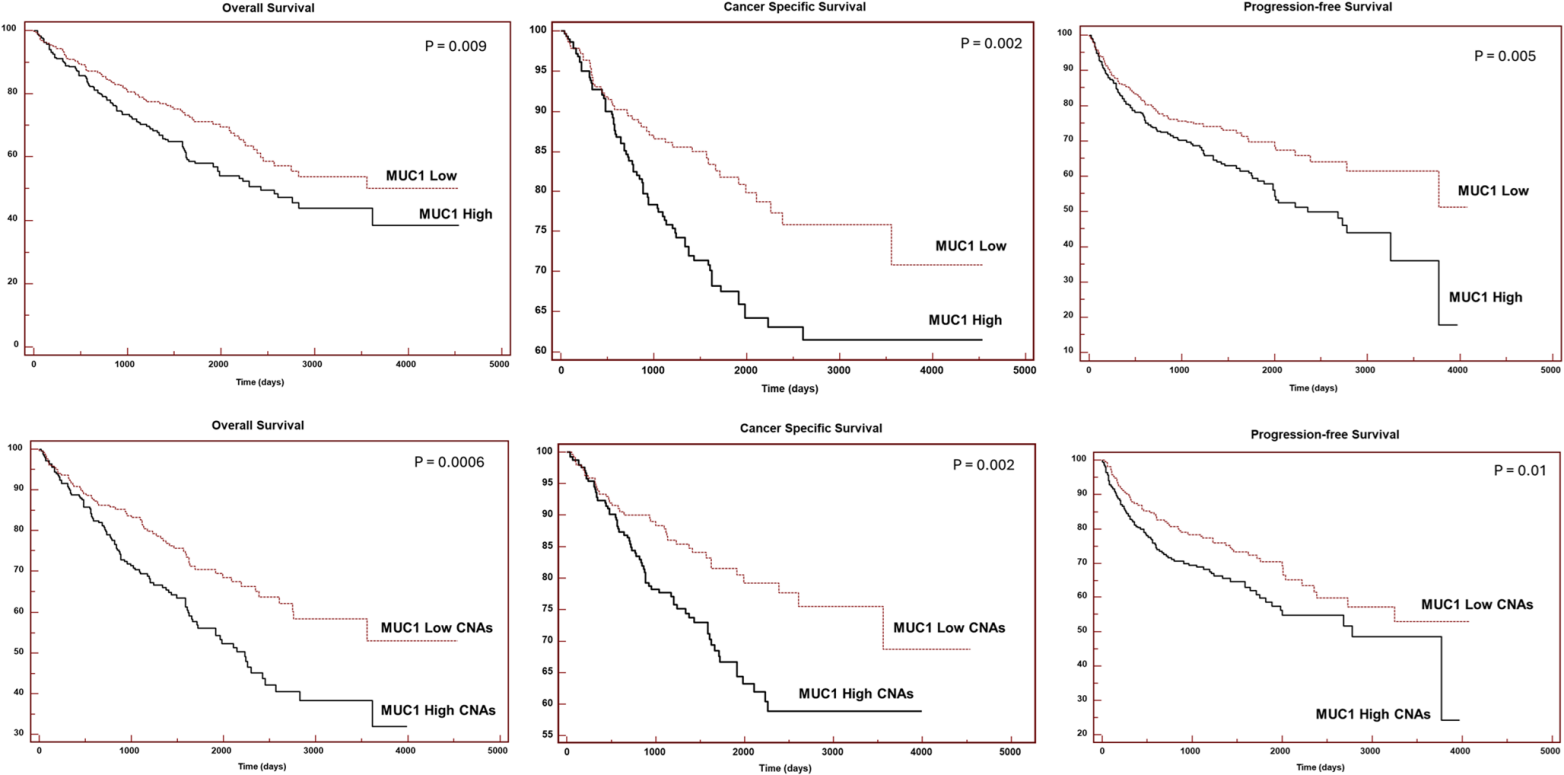
Prognostic role of MUC1 expression and gene copy number alterations (CNAs) in terms of overall survival, cancer specific survival, and progression-free survival in KIRC-TCGA database

Next, to investigate in vivo the proangiogenic capabilities of tumor cells alone or treated with sunitinib, cell suspensions were seeded on the top of a chick embryo chorioallantoic membrane (CAM) and their ability to induce vessel formation was evaluated. As shown in Fig. 4C, MUC1^H^ cancer cells were surrounded by a higher number of allantoic vessels developing radially toward them in a ‘spoked wheel’ pattern, compared to MUC1^L^ cells. But when MUC1^H^ cells were incubated with sunitinib, they induced a lower vascular reaction, evaluated as reduced vascular area and lower number of vessels branching points (Figs. 4D and 5E).

**Fig. 6:**
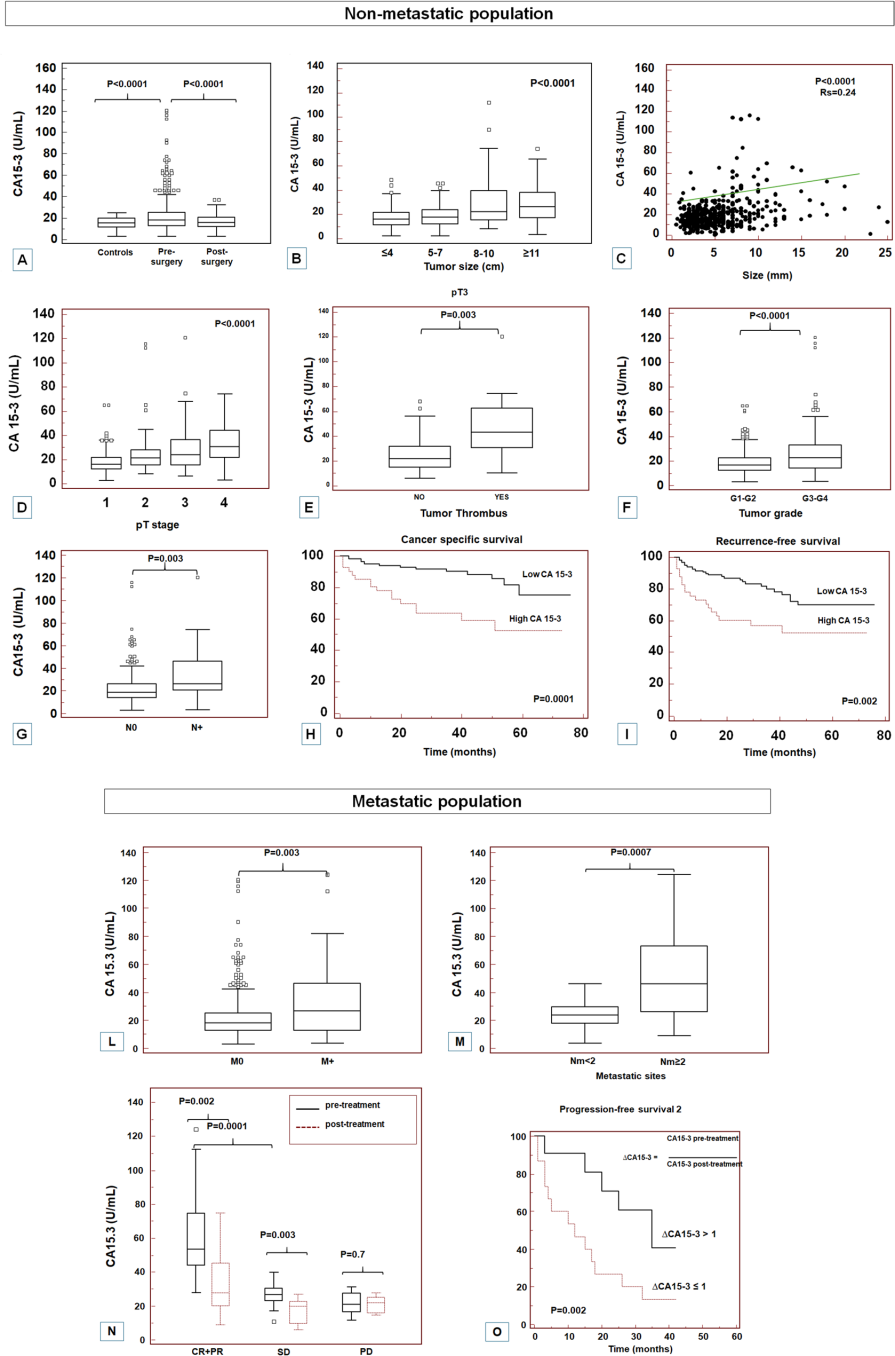
Association between CA15-3 serum levels and effects of surgery (**A**), tumor size (**B**-**C**), tumor stage (**D**), presence of tumor thrombus in pT3 patients (**E**), pathological grade (**F**), and presence of lymph node metastases (**G**) in non-metastatic population. Kaplan-Meier curves for cancer specific survival (**H**) and recurrence-free survival (**I**), stratified by serum CA15-3 levels in non-metastatic population. Association between CA15-3 serum levels and presence of visceral metastases (**L**), and number of metastatic sites (**M**). CA15-3 serum levels pre- and post-treatment in metastatic patients for achieving CR + PR, SD, and PD after 24 weeks of sunitinib administration (**N**). Kaplan-Meier curve for progression-free survival 2 stratified by ΔCA 15 − 3 ratio (**O**)

### MUC1 is a survival-associated gene marker for ccRCC

The prognostic role of MUC1 in KIRC-TCGA cohort was evaluated using Kaplan Meier curves. As shown in Fig. 6, patients with high MUC1 expression had poor OS (*P* = 0.009), CSS(*P* = 0.002), and PFS (*P* = 0.005). Similarly, a high frequency of CNAs predicted a worse prognosis in ccRCC patients.

#### Association between CA15-3 serum levels and clinicopathological characteristics in non-metastatic patients

Detailed clinical and pathological characteristics of these patients are summarized in Supplementary Table 2. Spearman’s test showed a positive correlation between MUC1 tissue expression and CA15-3 serum levels (rs = 0.68; *P* = 0.0001; Supplementary Fig. 5).

At the time of nephrectomy, 31% (*n* = 284) of the patients had abnormal levels (i.e., above the upper limit of the reference range) of CA 15 − 3. One month after surgery, CA15-3 serum levels were significantly reduced (*P* < 0.0001, Fig. 5A).

Statistically significant differences resulted between CA 15 − 3 values and tumor size (*P* < 0.0001; Spearman’s correlation: Rs = 0.24; *P* < 0.0001), tumor stage (*P* < 0.0001), presence of tumor thrombus in pT3 patients (*P* = 0.002), pathological grade (G1-2 vs. G3-4) (*P* < 0.0001), and presence of lymph node metastases (N0 vs. N+) (*P* = 0.003) (Fig. 5B-G). These results were further confirmed by Spearman’s correlation comparing CA15-3 values pT stage (*P* < 0.0001, Rs = 0.30) and grade (*P* = 0.0001, Rs = 0.21).

#### Cancer-specific survival in non-metastatic population

Kaplan-Meier survival curves for CSS, stratified by serum CA15-3 levels are shown in Fig. 5H. CSS was significantly decreased for patients with high levels of CA 15 − 3 (*P* = 0.0001). Univariate analyses for the predefined variables showed that pathological stage, presence of nodal metastases, pathological grade, tumor size, presence of necrosis and high levels of CA 15 − 3 were significantly associated with the risk of death. Multivariate analysis by Cox regression modeling confirmed that high CA 15 − 3 levels were an independent adverse prognostic factor for CSS (Table 1).

**Table 1:** Univariate and multivariate analyses for cancer-specific survival and recurrence-free survival

Variable	Category	Cancer-specific survival							
		Univariate Analysis				Multivariate Analysis			
		HR	CI 95%		*p* value	HR	CI 95%		*p* value
			Lower	Higher			Lower	Higher	
T stage	T3-4 vs. T1-2	2.04	1.45	2.36	***0.0001***	1.32	1.11	2.05	***0.001***
N stage	N + vs. N0	2.66	1.91	5.23	***0.001***	2.15	1.18	3.91	***0.01***
Grade	G3-4 vs. G1-2	2.12	1.11	5.34	***0.01***	1.56	1.12	2.28	***0.01***
Necrosis	Yes vs. No	2.32	1.19	3.58	***0.001***	1.73	1.36	2.28	***0.01***
Tumor size	Continuous	1.47	1.13	2.22	***0.01***	-	-	-	***-***
CA15-3	> 25 vs. ≤25 U/mL	2.16	1.19	5.41	***0.001***	1.94	1.02	2.86	***0.01***

Variable	Category	Recurrence-free survival							
		Univariate Analysis				Multivariate Analysis			
		HR	CI 95%		*p* value	HR	CI 95%		*p* value
			Lower	Higher			Lower	Higher	
T stage	T3-4 vs. T1-2	2.21	1.32	2.84	***0.001***	1.92	1.19	2.75	***0.001***
N stage	N + vs. N0	2.84	1.91	6.33	***0.001***	1.85	1.12	2.68	***0.01***
Grade	G3-4 vs. G1-2	2.06	1.88	5.22	***0.01***	1.61	1.01	3.79	***0.01***
Necrosis	Yes vs. No	2.17	1.12	3.27	***0.001***	1.96	1.21	2.68	***0.01***
Tumor size	Continuous	1.96	1.11	2.74	***0.01***	-	-	-	***-***
CA15-3	> 25 vs. ≤25 U/mL	2.15	1.05	4.54	***0.001***	1.93	1.12	2.92	***0.01***

**Table 2:** Univariate and multivariate analyses for cancer-specific survival and recurrence-free survival

Variable	Category	Univariate Analysis				Multivariate Analysis			
		HR	CI 95%		*p* value	HR	CI 95%		*p* value
			Lower	Higher			Lower	Higher	
T stage	T3-4 vs. T1-2	1.22	1.06	3.21	***0.01***	-	-	-	***-***
Grade	G3-4 vs. G1-2	2.16	1.42	3.26	***0.001***	1.71	1.11	3.68	***0.01***
Necrosis	Yes vs. No	1.97	1.21	4.23	***0.001***	1.92	1.20	2.98	***0.01***
Tumor size	Continuous	1.66	1.01	3.64	***0.01***	-	-	-	***-***
ΔCA15-3	≤ 1 vs. > 1	2.55	1.25	4.73	***0.001***	1.97	1.22	2.89	***0.01***

CI: confidence interval; HR: hazard ratio

#### Recurrence-free survival in non-metastatic population

Kaplan-Meier survival curves for RFS, stratified by serum CA15-3 levels, are shown in Fig. 6I. RFS was significantly shorter for patients with high levels of CA 15 − 3 (*P* = 0.002). Univariate analyses for the predefined variables showed that pathological stage, presence of nodal metastases, pathological grade, tumor size, presence of necrosis and high CA 15 − 3 levels were significantly associated with the risk of disease recurrence. In multivariate analysis, elevated CA15-3 was an independent risk factor for RFS (Table 1).

### Clinical response to sunitinib in metastatic population

In silico, in vitro and in vivo experiments demonstrated how MUC1^H^ cancer cells when treated with sunitinib had a greater death rate and a reduced capability to induce angiogenesis compared to MUC1^L^ tumor cells.

To translate these results into clinical practice, we evaluated the response to TKIs in patients with metastatic ccRCC who received sunitinib for at least 24 weeks.

Detailed clinical and pathological characteristics of these patients are summarized in Supplementary Table 3. Patients with metastatic disease had increased levels of CA15-3 compared to non-metastatic population (*P* = 0.003) (Fig. 6L). Moreover, patients with more than 2 metastatic sites had higher serum levels of CA15-3 compared to patients with metastasis in ≤ 2 organs (*P* = 0.007) (Fig. 6M).

All patients were clinically evaluated for treatment response after 24 weeks of sunitinib administration. The pre-treatment median level of CA 15 − 3 for patients achieving CR + PR was 53.5 U/mL and significantly reduced to 28 U/mL after treatment (*P* = 0.002). Similar findings were observed also in patients with SD (*P* = 0.003), but not in case of PD (*P* = 0.7) (Fig. 6N).

In addition, patients who experienced a reduction in CA15-3 levels after therapy (ΔCA 15 − 3 > 1), had a longer PFS2 than patients with no decrease or increase in values (*P* = 0.002) (Fig. 6O). In multivariate analysis, ΔCA 15 − 3 ≤ 1 was an independent risk factor for PFS2 (Table 2).

### Differential metabolomic analysis between sunitinib responders and non-responders according to MUC1 expression

**Fig. 7:**
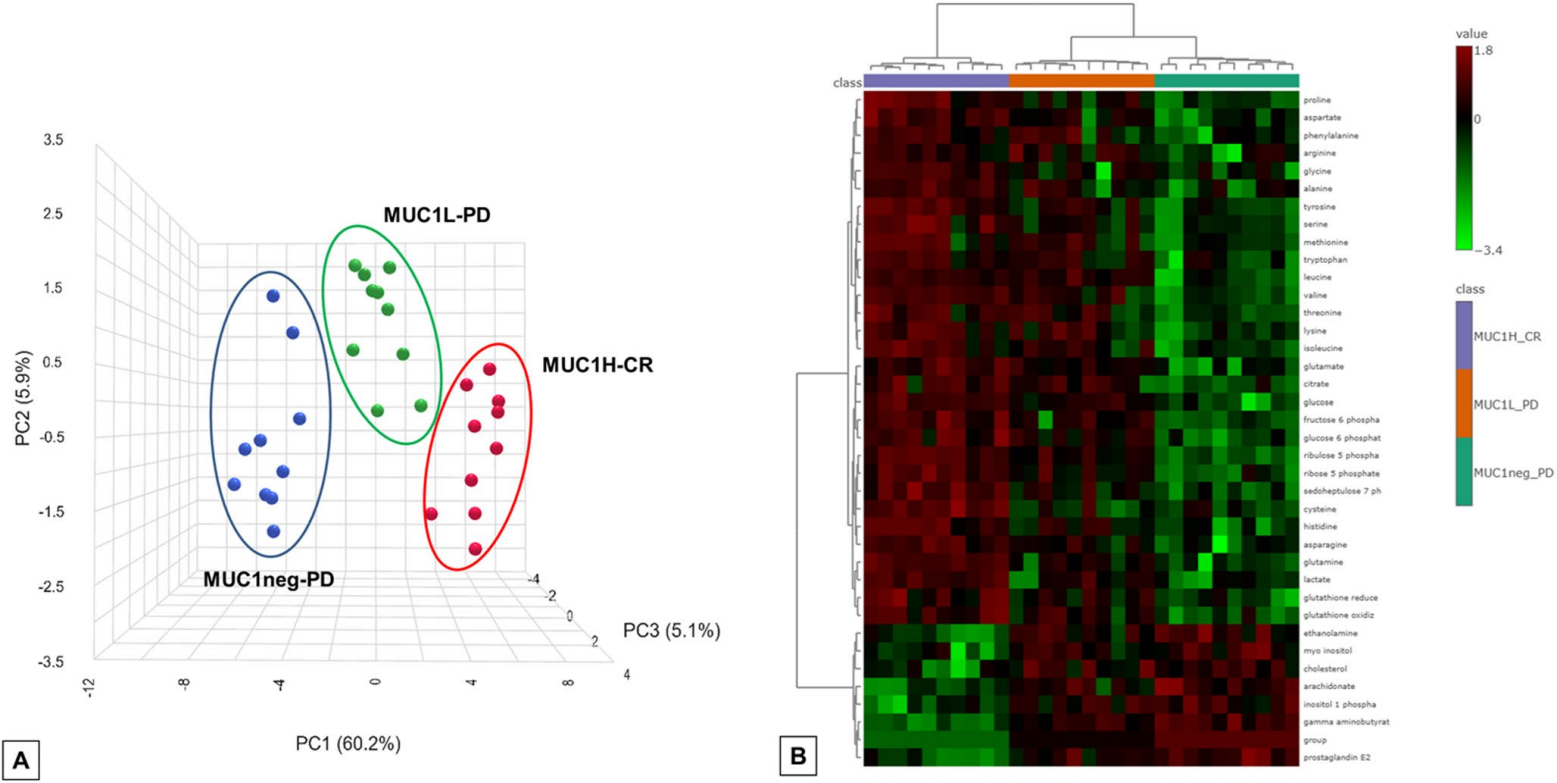
Principal component analysis (PCA) (**A**) and hierarchical clustering heatmap (**B**) to differentiate MUC1^H^-CR, MUC1^L^-PD and MUC1^neg^-PD samples as a function of the global tissue metabolome

To evaluate the MUC1-associated metabolic reprogramming in sunitinib responsive patients, untargeted metabolomic analysis was performed on 30 primary ccRCC derived from metastatic patients treated with sunitinib, and including MUC1^H^-CR (*n* = 10), MUC1^L^-PD (*n* = 10), and MUC1^neg^-PD. The application of hierarchical clustering and PCA to differentiate MUC1^H^-CR from MUC1^L^-PD and MUC1^neg^-PD samples as a function of the global tissue metabolome showed that these three groups were clearly distinguishable (Fig. 7A and B).

**Fig. 8:**
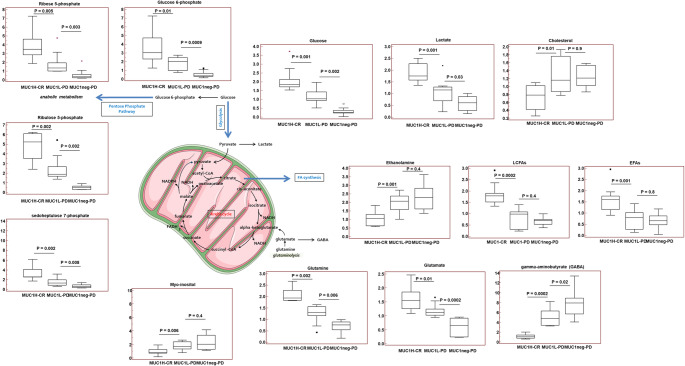
Schematic model summarizing the differences in glucose and lipid metabolism between MUC1^H^-CR, MUC1^L^-PD and MUC1^neg^-PD tumor tissues. Y-axis: metabolite relative amount

MUC1^H^-CR tumors showed increased accumulation of glycolytic metabolites and high levels of lactate compared to PD tumors, indicating activation of the Warburg effect. Metabolic intermediates related to pentose phosphate pathway were also increased in MUC1^H^-CR tumors, suggesting an increased activation of this pathway for anabolic reactions (Fig. 8).

**Fig. 9:**
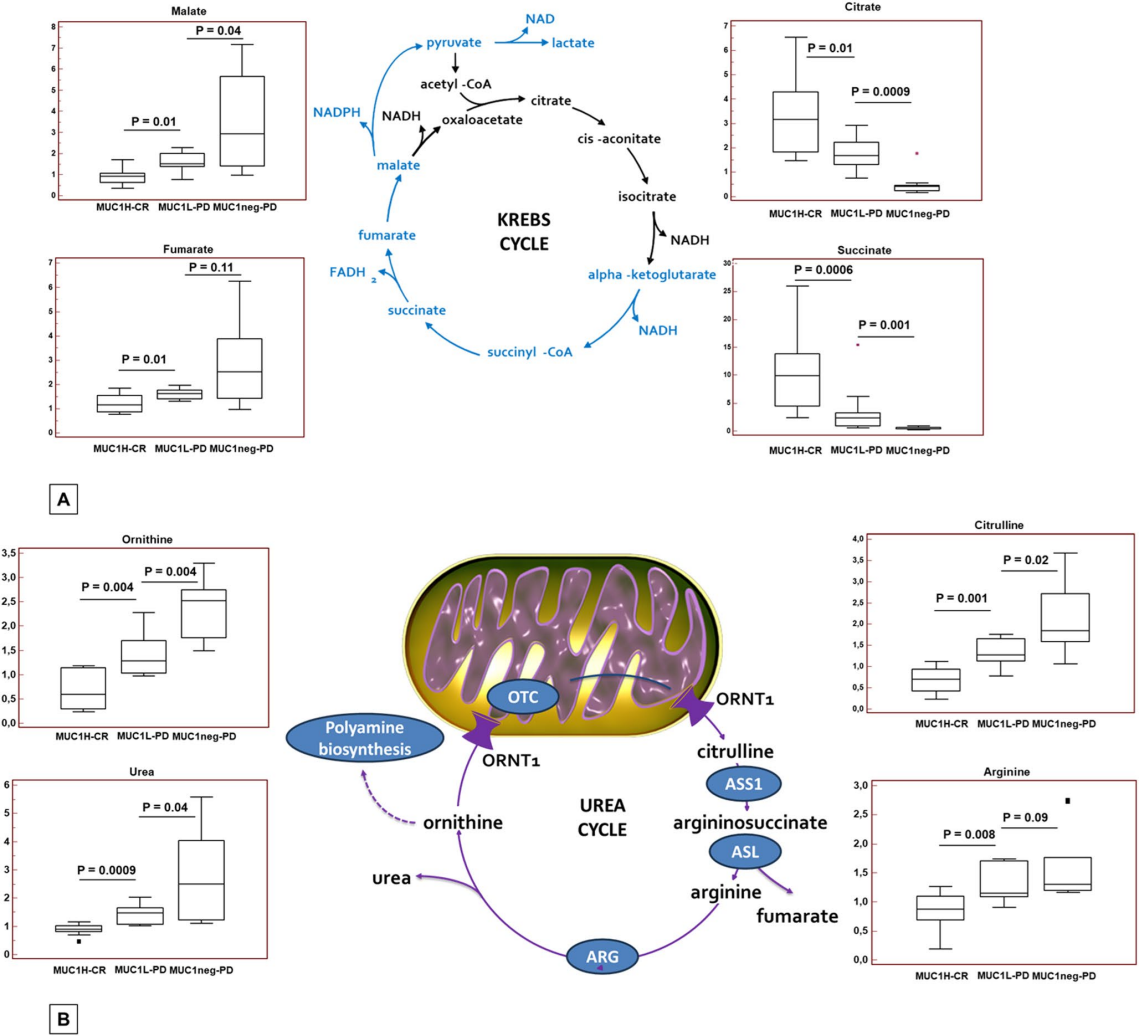
Schematic model summarizing the differences in tricarboxylic acid cycle (**A**) and urea cycle (**B**) metabolites between MUC1^H^-CR, MUC1^L^-PD and MUC1^neg^-PD tumor tissues. Y-axis: metabolite relative amount. ASL: Argininosuccinate lyase. ASS1: Argininosuccinate synthase 1. ARG: Arginase. ORNT1: Ornithine transporter 1. OTC: Ornithine transcarbamylase.

Analysis of tricarboxylic acid (TCA) cycle metabolites showed that citrate and succinate levels were increased in MUC1^H^-CR cancer tissues whereas fumarate and malate were significantly reduced when compared to MUC1^L^-PD and MUC1^neg^-PD tumors (Fig. 9A). These findings, in association with increased expression of NDUFA4L2 and reduced number of mitochondria (Fig. 3D), indicate a downregulation of oxidative phosphorylation and mitochondrial bioenergetics in MUC1^H^ tumors. Since the TCA cycle and the urea cycle are linked via the asparatate/argininosuccinate shunt, we next analyzed Krebs cycle metabolites. We found reduced levels of urea, citrulline and ornithine in MUC1^H^-CR tumors compared to MUC1^L^-PD and MUC1^neg^-PD renal cancers (Fig. 9B). This down-regulation in ammonia metabolism observed in MUC1^H^-CR ccRCC, aims to maintain elevated levels of pyridoxal-5’-phosphate for amino acid homeostasis and to avoid the toxic accumulation of polyamines, as previously described [ref].

Alteration of lipid metabolism is another key feature of ccRCC. We observed the accumulation of many essential and long-chain fatty acids in responder group (Supplementary Table 4), while non-responder groups showed increased levels of metabolites related to glycerolipid (including ethanolamine and phosphoethanolamine) and eicosanoid metabolism (arachidonate, prostaglandin E2, 5-HETE, and 5-oxoETE). MUC1^L^-PD and MUC1^neg^-PD tumors, were characterized also by increased levels of all the free amino acids except for glutamate, glutamine and cysteine. Interestingly, non-responder groups showed an increased accumulation of gamma-aminobutyrate (GABA), cholesterol and inositol metabolism intermediates (myo-inositol, inositol 1-phosphate, and chiro-inositol).

**Fig. 10:**
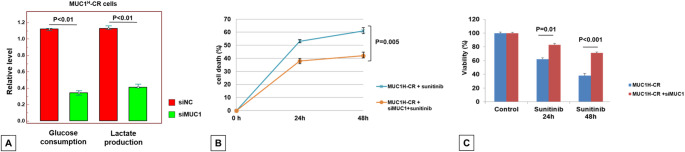
Analysis of glucose consumption and lactate production in MUC1^H^-CR cells treated with MUC1 siRNA or negative control (NC) (**A**). The data represent the means ± s.d. of three independent experiments. Death rate (**B**) and MTT assay (**C**) of MUC1^H^ -CR tumor cells treated with MUC1 siRNA or negative control and sunitinib

Overall, these findings suggested that MUC1 overexpression, through induction of the Warburg effect, increased the vulnerability of renal cancer cells to sunitinib treatment. To investigate this mechanism, MUC1^H^-CR ccRCC cells were treated with siMUC1 (Supplementary Fig. 2) and both glucose consumption and lactate production were evaluated. MUC1 depletion significantly reduced glucose consumption and lactate production (Fig. 10A) and increased the resistance of cancer cells to sunitinib administration (Fig. 10B and C).

## Discussion

In this study, we comprehensively explored the expression and clinical role of MUC1 and its soluble form CA15-3 in ccRCC, integrating transcriptomic, metabolomic, immunohistochemical, and clinical data. Single-cell transcriptomic analysis showed that MUC1 was predominantly expressed in malignant epithelial cells, with significant associations with key oncogenic features, such as angiogenesis, proliferation, stemness, and hypoxia. These findings were confirmed by gene set enrichment analysis that revealed a strong enrichment of hallmark pathways related to EMT, metabolic reprogramming, complement activation, and inflammatory signaling in MUC1^H^ compared to MUC1^L^ renal cancer. Histological and immunofluorescence evaluation of MUC1^H^ tumors revealed a higher MVD, activation of EMT program and increased expression of metabolic adaptation markers (elevated NDUFA4L2 levels, and decreased mitochondrial mass), all consistent with a more aggressive tumor phenotype. In addition, the co-localization of C3 and PTX3 confirmed the activation of complement system on tumor site and immune microenvironment modulation of MUC1-driven renal cancer, as previously described [32–35].

In clinical setting, MUC1 overexpression and genomic alterations were associated with significantly worse overall survival, cancer-specific survival, and recurrence-free survival confirming MUC1 as a survival-related prognostic factor in ccRCC. These findings highlight the value of MUC1 in risk stratification, particularly when combined with histological and clinicopathological parameters. CA15-3 serum levels were elevated in about one-third of patients with localized ccRCC and correlated positively with tumor burden, grade, stage, and adverse pathological features. Interestingly, CA15-3 levels significantly decreased post-nephrectomy, reinforcing their tumor-derived origin. In addition, high preoperative CA15-3 levels were independently associated with shorter cancer-specific survival and recurrence-free survival, as shown by Kaplan-Meier curves and multivariate analysis.

Clear cell renal carcinoma is one of the most chemoresistant tumors, and in the past decades, cytoreductive nephrectomy has been a milestone in the management of metastatic renal cancer [36, 37]. In subsequent years, an in-depth understanding of the molecular biology of RCC has led to introduction of anti-angiogenic drugs for the treatment of metastatic tumor, although these molecules yield complete or partial responses in a minority of patients. Recently, the use of ICIs has revolutionized the therapeutic approach for metastatic RCC, although resistance to these agents, both innate and acquired, remains a challenge [17]. In a previous study we identified MUC1-expressing tumors as an immune-silent subgroup of ccRCC, characterized by low immune infiltration, low expression of PD-L1, high M2-like tumor-associated macrophages response, and metabolic reprogramming [10, 11]. Based on these characteristics we hypothesized that MUC1^H^ ccRCC could be more responsive to antiangiogenic therapies rather than ICIs. Effectively, in silico and in vitro data suggest that MUC1 overexpression may confer increased sensitivity to sunitinib. Therefore, to test this hypothesis in vivo, we first evaluated the ability MUC1^H^ tumor cells to induce an angiogenic response compared to MUC1^L^ cells, using a CAM assay. Next, we evaluated the response to sunitinib treatment in terms of inhibition of angiogenesis in relation to MUC1 expression.

Overall, our results showed that MUC1^H^ cancer cells induced an increased angiogenesis compared to MUC1^L^ cancer cells, although MUC1-expressing tumor cells were more sensitive to antineoplastic effects of sunitinib than MUC1^L^ cells.

Clinically, patients with higher baseline CA15-3 levels were more likely to achieve a therapeutic response with sunitinib. Moreover, a post-treatment reduction in CA15-3 levels (ΔCA15-3 > 1) was strongly associated with prolonged progression-free survival, highlighting its potential as a dynamic marker for monitoring treatment efficacy.

Considering the activity of MUC1 as metabolic regulator, to better understand its role in modulating the sunitinib sensitivity in the context of metabolic reprogramming, we comprehensively analyzed the metabolomic signature of responders versus non-responders’ tumors. Moreover, to better highlight the differences in metabolic pathways, we selected primary tumor samples derived from patients that showed a CR versus PD and stratified the tissues according to MUC1 expression.

MUC1^H^-CR tumors were characterized by metabolic changes involving glucose and lipid metabolism, and these alterations in association with impaired oxidative phosphorylation suggested activation of the Warburg effect. In accordance with these results, Zhang et al. demonstrated that ccRCC cells with increased glycolytic activity were more vulnerable to sunitinib treatment [38]. MUC1^L^-PD and MUC1^neg^-PD tumors showed instead accumulation of all amino acids except glutamine, glutamate and cysteine. Moreover, specific metabolic perturbations in sunitinib-resistant tumors included alterations in urea, glycolipid, inositol and cholesterol pathways and increased accumulation of GABA.

These findings collectively suggest that MUC1/CA15-3 is a biological hallmark of tumor aggressiveness and may serve as a biomarker for prognostication and therapeutic stratification in ccRCC. Given the limited availability of reliable blood-based biomarkers in kidney cancer, CA15-3 presents an appealing candidate for integration into clinical decision-making.

Despite the strengths of our multi-modal approach, some limitations must be acknowledged. First, although the CA15-3 assay is widely available, its lack of specificity in non-malignant conditions (e.g., liver disease, inflammatory disorders, etc.) may limit its utility as a stand-alone biomarker in unselected populations. Second, while we performed a large analysis of non-metastatic patients, the metastatic cohort was relatively small and derived from a single institution. Larger, multicenter studies are needed to validate CA15-3 role in predicting treatment response. Third, although our in vitro and in vivo findings support a functional role of MUC1 in tumor progression and drug sensitivity, additional mechanistic studies are warranted to explore whether MUC1 directly modulates the efficacy of tyrosine kinase inhibitors or simply marks a more responsive tumor subtype.

## Conclusion

This study demonstrates that MUC1 overexpression and its soluble form CA15-3 are associated with aggressive biological characteristics of ccRCC. CA15-3 serum levels not only reflect tumor burden in localized disease but are independently associated with survival and recurrence. In the metastatic setting, MUC1/CA15-3 identifies a subset of tumors more sensitive to sunitinib, with elevated pre-treatment CA15-3 levels and their dynamic decrease during therapy correlating with better clinical outcomes. These results suggest the integration of serum CA15-3 into prognostic and predictive models for ccRCC management. Future multicenter prospective trials and mechanistic investigations are warranted to validate CA15-3 as a biomarker and to clarify MUC1 direct role in modulating response to anti-angiogenic therapy.

## Supplementary Information

Below is the link to the electronic supplementary material.

Supplementary Material 1

Supplementary Material 2

Supplementary Material 3

Supplementary Material 4

Supplementary Material 5

Supplementary Material 6

Supplementary Material 7

Supplementary Material 8

Supplementary Material 9

## Data Availability

The datasets used and/or analyzed during the current study are available from the corresponding author upon reasonable request. The datasets used in this study are openly available at: [http://biocc.hrbmu.edu.cn/CancerSEA/](http:/biocc.hrbmu.edu.cn/CancerSEA)[https://xena.ucsc.edu/](https:/xena.ucsc.edu)[https://github.com/openbiox/UCSCXenaShiny](https:/github.com/openbiox/UCSCXenaShiny)[https://www.ncbi.nlm.nih.gov/geo/query/acc.cgi? acc=GSE15641](https:/www.ncbi.nlm.nih.gov/geo/query/acc.cgi? acc=GSE15641).

## Abbreviations

CAM: chick embryo chorioallantoic membrane
ccRCC: clear cell-renal cell carcinoma
CNAs: copy number alterations
CR: complete response
CSS: cancer-specific survival
EMT: epithelial–mesenchymal transition
GABA: gamma-aminobutyrate
GC: Gas Chromatography
LC: Liquid chromatography
MS: Mass Spectrometry
MTT: 3-(4,5-dimethylthiazol-2-yl)-2,5-diphenyltetrazolium bromide
MVD: microvessel density
PFS: progression-free survival
PD: progressive disease
PR: partial response
RFS: recurrence-free survival
SD: stable disease
