# Supplementary figures and images for: MUC1/CA15-3 identifies a clear cell renal carcinoma characterized by Sunitinib response with a specific metabolic signature
Source: Clin Exp Med. 2026 Jan 14;26(1):106. doi: 10.1007/s10238-026-02042-5 (PMC12819446; doi:10.1007/s10238-026-02042-5)

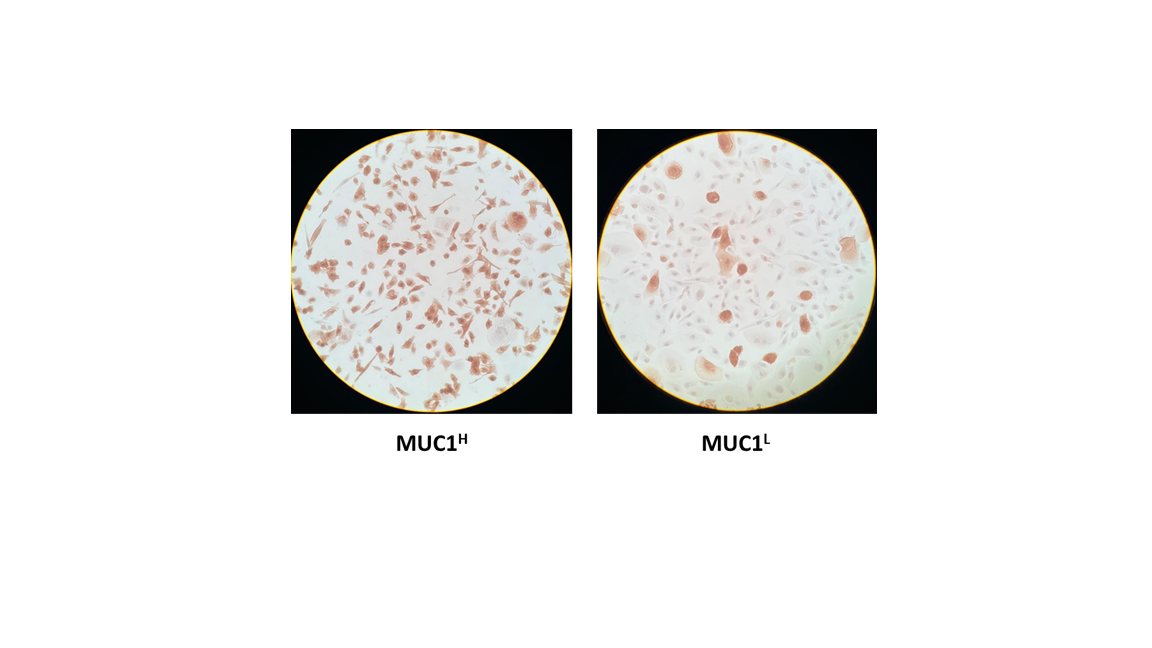

Supplement: Supplementary file 1 — Supplementary Material 1 [file 10238_2026_2042_MOESM1_ESM.tif]

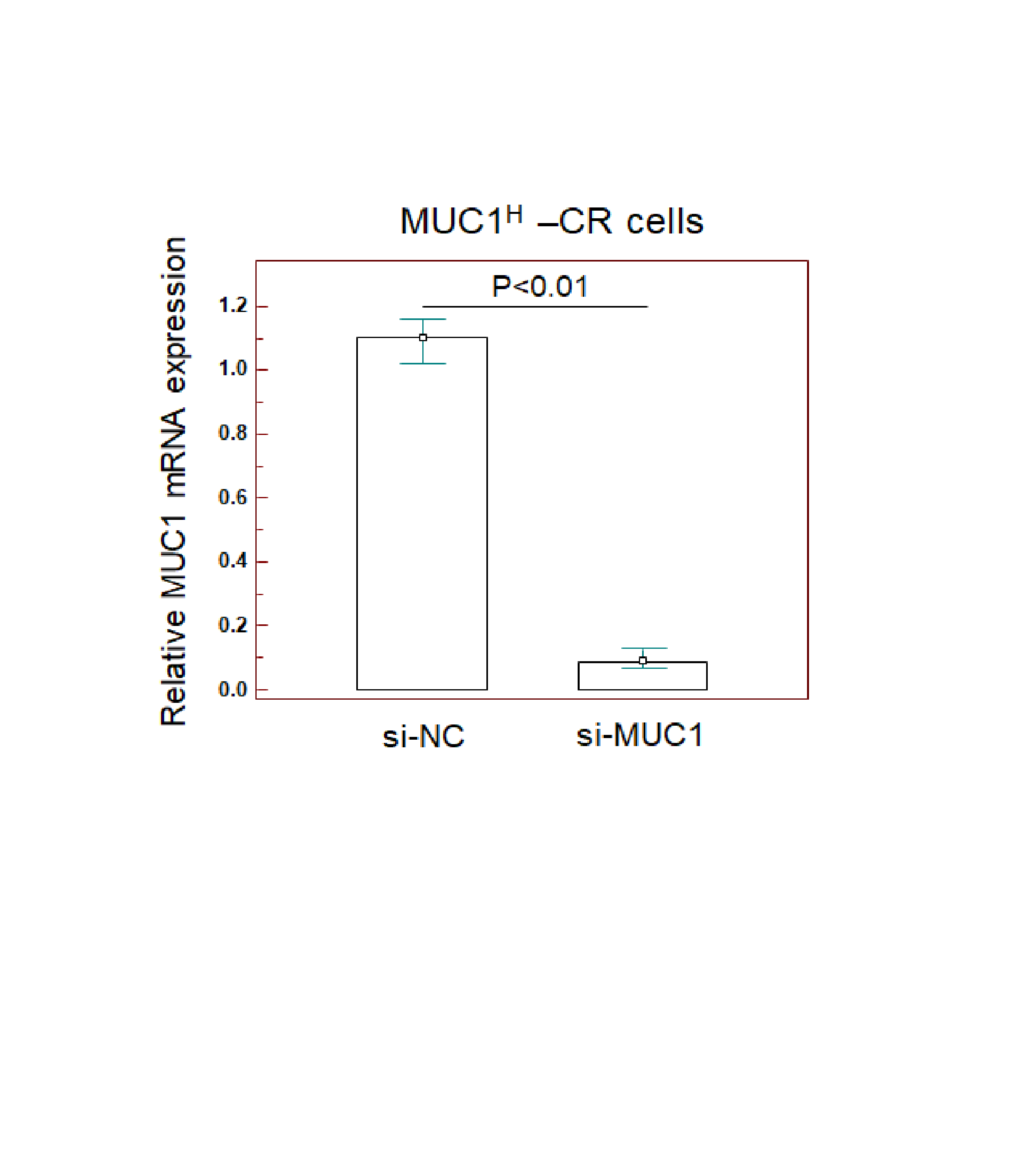

Supplement: Supplementary file 2 — Supplementary Material 2 [file 10238_2026_2042_MOESM2_ESM.tif]

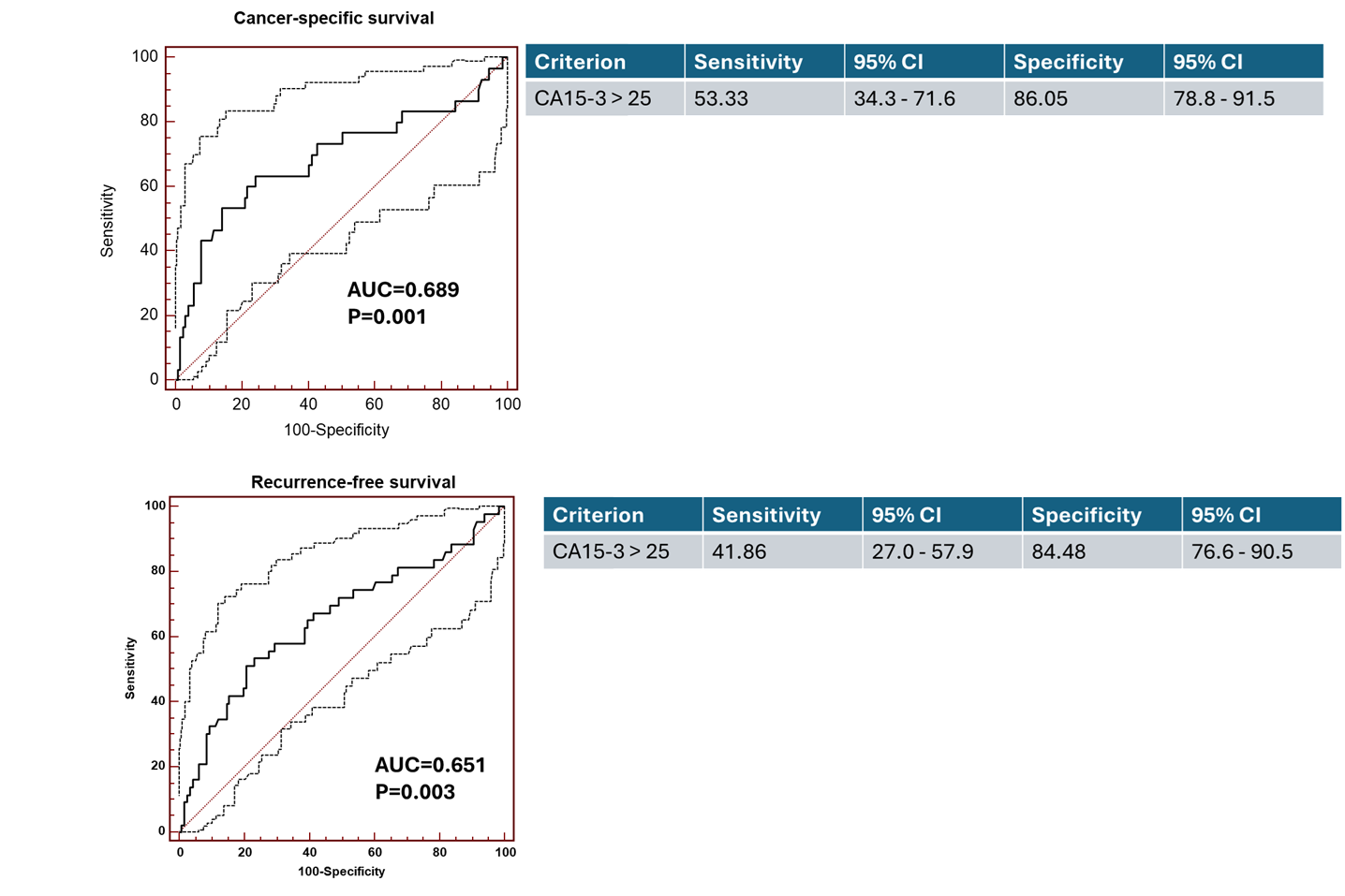

Supplement: Supplementary file 3 — Supplementary Material 3 [file 10238_2026_2042_MOESM3_ESM.tif]

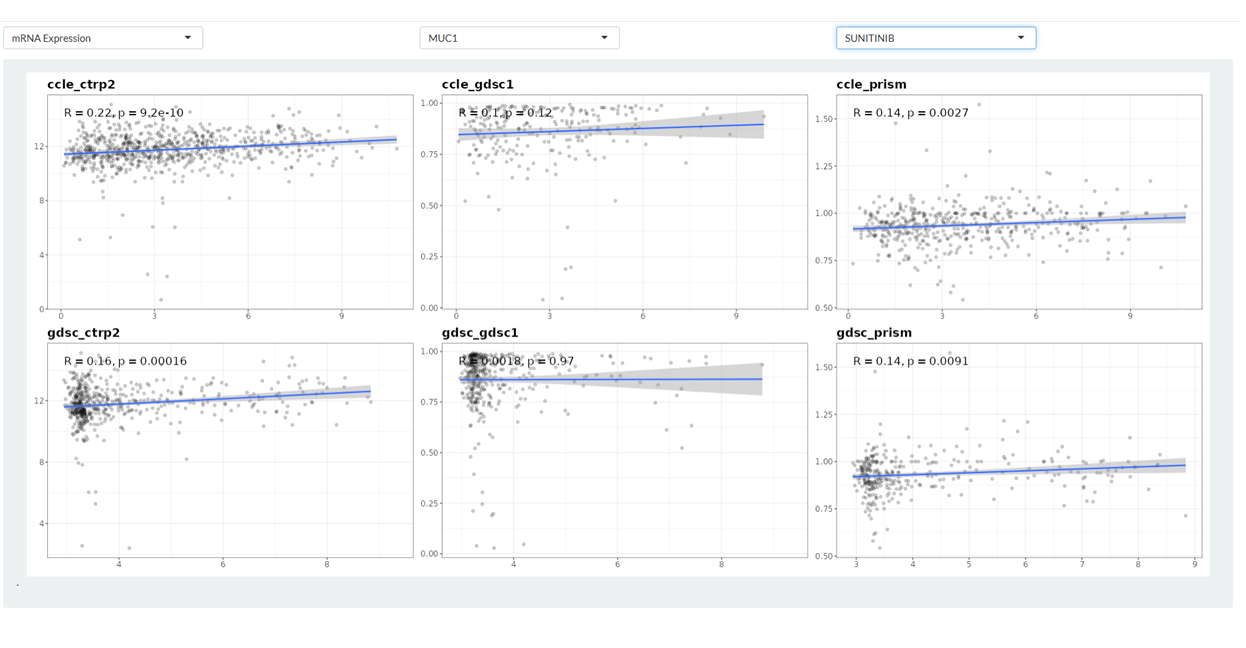

Supplement: Supplementary file 4 — Supplementary Material 4 [file 10238_2026_2042_MOESM4_ESM.tif]

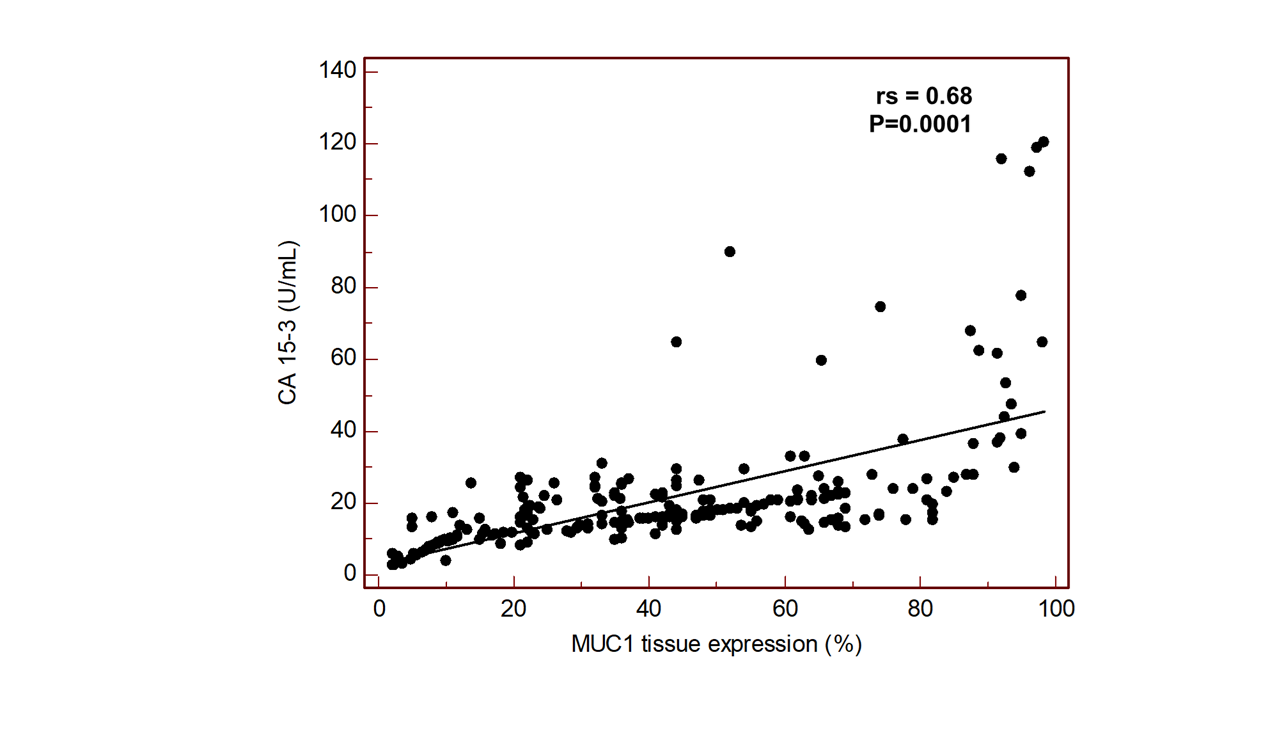

Supplement: Supplementary file 5 — Supplementary Material 5 [file 10238_2026_2042_MOESM5_ESM.tif]
