# Supplementary material for: MUC1/CA15-3 identifies a clear cell renal carcinoma characterized by Sunitinib response with a specific metabolic signature
Source: Clin Exp Med. 2026 Jan 14;26(1):106. doi: 10.1007/s10238-026-02042-5 (PMC12819446; doi:10.1007/s10238-026-02042-5)
Supplement: Supplementary file 6 — Supplementary Material 6 [file 10238_2026_2042_MOESM6_ESM.docx]

| MUC1 | 5′-GAACTACGGGCAGCTGGACATC-3′  5′-GCTCTCTGGGCCAGTCCTCCTG-3′ |
| --- | --- |
| B-ACTIN | 5’-AATCTGGCACCACACCTTCT-3’  5’-AGCCTGGATAGCAACGTACA-3’ |

Supplementary Table 1: Primers used for real time PCR
