# Supplementary material for: MUC1/CA15-3 identifies a clear cell renal carcinoma characterized by Sunitinib response with a specific metabolic signature
Source: Clin Exp Med. 2026 Jan 14;26(1):106. doi: 10.1007/s10238-026-02042-5 (PMC12819446; doi:10.1007/s10238-026-02042-5)
Supplement: Supplementary file 7 — Supplementary Material 7 [file 10238_2026_2042_MOESM7_ESM.doc]

| **Variable** | **n=914** |
| --- | --- |
| **Age (years)**  **median**  **95% CI** | 64  63-65 |
| **Gender**  **Male**  **Female** | 565 (61.8%)  349 (38.2%) |
| **Dimensions (cm)**  **median**  **95% CI** | 4.3  4.0 - 4.6 |
| **Pathological stage**  **pT1**  **pT2**  **pT3**  **pT4** | 596 (65.2%)  196 (21.5%)  94 (10.3%)  28 (3%) |
| **pN+** | 76 (8.3%) |
| **Fuhrman grade**  **G1-2**  **G3-4** | 528 (57.8%)  386 (42.2%) |
| **CA15-3 (U/mL)**  **Median**  **95% CI** | 18.1  17.1 – 19.2 |

**Supplementary Table 2:** Clinical and pathological characteristics of non-metastatic ccRCC patients enrolled for CA15-3 levels evaluation
