# Supplementary material for: MUC1/CA15-3 identifies a clear cell renal carcinoma characterized by Sunitinib response with a specific metabolic signature
Source: Clin Exp Med. 2026 Jan 14;26(1):106. doi: 10.1007/s10238-026-02042-5 (PMC12819446; doi:10.1007/s10238-026-02042-5)
Supplement: Supplementary file 8 — Supplementary Material 8 [file 10238_2026_2042_MOESM8_ESM.doc]

| **Variable** | **n=48** |
| --- | --- |
| **Age (years)**  **median**  **95% CI** | 59  55-65 |
| **Gender**  **Male**  **Female** | 30 (62.5%)  18 (37.5%) |
| **Pathological stage**  **pT1**  **pT2**  **pT3**  **pT4** | 4 (8.3%)  8 (16.7%)  28 (58.3%)  8 (16.7%) |
| **Tumor grade**  **G1-2**  **G3-4** | 13 (27%)  35 (73%) |
| **CA15-3 (U/mL)**  **Median**  **95% CI**  **range** | 26  23 – 28  6 – 124 |

**Supplementary Table 3:** Clinical and pathological characteristics of metastatic ccRCC patients
