# Supplementary material for: MUC1/CA15-3 identifies a clear cell renal carcinoma characterized by Sunitinib response with a specific metabolic signature
Source: Clin Exp Med. 2026 Jan 14;26(1):106. doi: 10.1007/s10238-026-02042-5 (PMC12819446; doi:10.1007/s10238-026-02042-5)
Supplement: Supplementary file 9 — Supplementary Material 9 [file 10238_2026_2042_MOESM9_ESM.docx]

**Essential fatty acids (EFAs)**

dihomo-linolenate (20:3n3 or n6)

docosahexaenoate (DHA; 22:6n3)

docosapentaenoate (n3 DPA; 22:5n3)

docosapentaenoate (n6 DPA; 22:5n6)

eicosapentaenoate (EPA; 20:5n3)

linoleate (18:2n6)

linolenate [alpha or gamma; (18:3n3 or 6)]

**Long chain fatty acids (LCFAs)**

10-heptadecenoate (17:1n7)

10-nonadecenoate (19:1n9)

adrenate (22:4n6)

arachidate (20:0)

arachidonate (20:4n6)

behenate (22:0)

cis-vaccenate (18:1n7)

dihomo-linoleate (20:2n6)

docosadienoate (22:2n6)

docosatrienoate (22:3n3)

eicosenoate (20:1n9 or 11)

erucate (22:1n9)

margarate (17:0)

myristate (14:0)

myristoleate (14:1n5)

nervonate (24:1n9)

nonadecanoate (19:0)

oleate (18:1n9)

palmitate (16:0)

palmitoleate (16:1n7)

pentadecanoate (15:0)

stearate (18:0)

stearidonate (18:4n3)

Supplementary Table 4: Detailed list of metabolites identified in EFAs and LCFAs categories
